# In vivo embryo development in bitches inseminated laparoscopically after ovulation time estimated based on a single progesterone determination

**DOI:** 10.1590/1984-3143-AR2022-0079

**Published:** 2023-03-06

**Authors:** Aracelle Elisane Alves, Tathiana Ferguson Motheo, Maricy Ferreira Apparicio, Giuliano Queiroz Mostachio, Ricarda Maria dos Santos, Wilter Ricardo Russiano Vicente, Gaia Cecilia Luvoni

**Affiliations:** 1 Departamento de Cirurgia Veterinária, Faculdade de Medicina Veterinária, Universidade Federal de Uberlândia, Uberlândia, MG, Brasil; 2 Programa de Pós-graduação Strictu Sensu em Biociência Animal, Universidade de Cuiabá, Cuiabá, MT, Brasil; 3 Departamento de Reprodução Animal, Faculdade de Medicina Veterinária e Zootecnia, Universidade Estadual Paulista, Botucatu, SP, Brasil; 4 Departamento de Cirurgia Veterinária, Faculdade de Medicina Veterinária, Centro Universitário de Rio Preto, Rio Preto, SP, Brasil; 5 Departamento de Reprodução Animal, Faculdade de Ciências Agrárias e Veterinárias, Universidade Estadual Paulista, Jaboticabal, SP, Brasil; 6 Dipartimento di Medicina Veterinaria e Scienze Animali, Università Degli Studi di Milano, Milano, Lombardia, Italia

**Keywords:** Intrauterine artificial insemination, embryo development, bitches

## Abstract

Logistic and economical limitations are often the causes of dog owners not accurately monitoring the estrous cycle and the optimal insemination time. The aim of this study was to evaluate *in vivo* early embryonic development in bitches, after the analysis of sequential vaginal cytologies associated to single progesterone measurement and single laparoscopic insemination with high quality semen (fresh and with high spermatozoa concentration) or low-quality semen (frozen/thawed and with low spermatozoa concentration) at 48 h post- ovulation time predicted on a single progesterone measurement. Ten bitches were inseminated with 250 x 10^6^ fresh spermatozoa (80% motility), and ten with 80 x 10^6^ frozen/thawed spermatozoa (60% motility) in the cranial part of each uterine horn. Seven days later, ovariohysterectomy was performed and the oviducts and uterine horns and body were flushed to recover embryos and unfertilized oocytes. In 80% of the bitches inseminated with fresh and 50% of bitches inseminated with frozen/thawed semen, embryos at 2 to 8 cells stage were recovered mostly from the, oviducts. This study indicates that pregnancies can be obtained with a single laparoscopic intrauterine insemination after single serum progesterone measurement, although with a low number of embryos. This result should be taken into account in case economic or logistic restrictions that affect the possibility of owners to plan an accurate monitoring of the optimal breeding time using fresh and frozen semen.

## Introduction

The precise determination of fertilization period is of great interest for successful reproduction in dogs, particularly when only one single mating or artificial insemination (AI) is performed.

The most striking feature of dog reproduction is the delay in the appearance of fertilizable oocytes after ovulation. Canine oocytes are ovulated on day 2-3 of LH surge as primary oocytes (Tsutsui, 1989; Mason, 2018) and meiosis occurs during descent in the oviducts.

In the oviducts, oocytes mature as secondary oocytes to be fertilized by spermatozoa approximately from four days until about seven days after the preovulatory LH surge (i.e. from two days until about five days after ovulation). Therefore, optimization of the chances for a bitch to become pregnant requires breeding or insemination during the fertilization period (England and Concannon, 2002). Assessment of the fertilization period is usually based on vaginal cytology and plasma or serum progesterone concentration, that rapidly increases from basal levels to 1-3 ng/mL during the preovulatory LH surge (Concannon, 2011; Antonov, 2017). In the bitch, the measurement of progesterone concentration during estrus is the most widely practiced methodology in clinical settings for timing AI, especially when frozen-thawed semen is used (Hollinshead and Hanlon, 2019). It has been reported that 48h before ovulation progesterone concentration ranges between 1 and 1.9 ng/mL, 24h before ovulation it ranges between 2 and 3.9 ng/mL while levels ranging from 4 to 10 ng/mL indicate the ovulation time (Johnston et al., 2001).

The evaluation of the gradual increase of progesterone requires serial measurements to estimate its trend and to improve the accuracy of the prediction of the fertilization period (Hollinshead and Hanlon, 2019; Macedo et al., 2012). However, repeated progesterone measurements increase the costs for the owner, and logistical and/or economic problems may limit animal movement. Thus, even though a continuous monitoring of the estrous cycle progression and more than one mating or insemination are usually recommended to increase fertility rates, it often happens that the owner has only one chance to detect the onset of estrus and to have the bitch pregnant. When only a single artificial insemination is planned, and particularly when cryopreserved semen is used, intrauterine rather than vaginal semen deposition increases fertility rates. This is achieved because the intrauterine artificial insemination (IUAI) avoids the reflux of semen and the difficult passage of spermatozoa through the cervix, and also the time taken by spermatozoa to reach the oocyte is shortened (Silva et al., 1996). In addition, as thawed semen has lower quality than fresh semen, due to the reduced survival of cryopreserved spermatozoa, the intrauterine insemination improves the chance to have the bitch pregnant (Mason, 2018).

Intrauterine insemination can be performed by transcervical endoscopy (Mason 2017), laparotomy (Silva et al., 1996) or laparoscopy (Valocký et al., 2003). Minimal invasive procedures as laparoscopy allows the visualization of the uterus and the deposition of semen on the cranial part of uterine horns, closer to the oviducts where fertilization takes place, thus potentially improving the chances of conception (Silva et al., 1995). To the best of our knowledge, *in vivo* embryo development after a single insemination timed on the basis of a single progesterone determination has not been described, despite the potential usefulness of the information for practitioner facing challenging situations and variable semen quality.

Hence, the present study evaluates the pregnancy rates and *in vivo* early embryonic development in bitches after a laparoscopic insemination with high or low quality semen (fresh semen with high concentration of spermatozoa or frozen-thawed semen with low concentration of spermatozoa) performed 48 h after the estimated ovulation time on the basis of a single progesterone determination.

## Methods

This study was conducted in accordance with federal legislation and was approved by the Animal Ethics Committee of São Paulo State University, protocol number 016781-07.

All chemicals were purchased from the Sigma Chemical Company (St. Louis, MO, USA), unless otherwise stated.

### Animals and estimation of ovulation time

Twenty adult, healthy, cross-breed bitches, aged 6 months to 5 years (mean ± SD: 2.7 ± 1.3 years; Table 1), nuliparous or pluriparous were used in this study. All animals were privately owned, and OVH was offered as a counterpart to the enrolment of the animals in the experiment. Owners consent was documented and signed in a specific owner consent form. Presence of vulvar discharge and edema were used as major clinical signs to determine the onset of proestrus. Vaginal cytology was daily performed at the clinic and stained with Panotic staining kit (Laborclin^®^, Pinhais, Brazil), the collection and analysis of the samples were performed by the same veterinary researcher and expert in this area. When vaginal cytology presented more than 90% cornified cells, the veterinary responsible for these examinations contacted by phone the owner requesting the presence of the animal for blood collection. One blood sample was collected by cephalic venipuncture for the measurement of serum progesterone concentration by chemiluminescence immunoassay (Volkmann, 2006). Ovulation time was estimated according to serum progesterone concentration as reported as follow: ovulation time was estimated in 48h when progesterone (P4) concentrations ranged between 1 and 1.9 ng/mL, in 24 h with progesterone ranging from 2 to 3.9 ng/mL, whereas levels ranging between 4 and 10 ng/mL indicated ovulation time (Johnston et al., 2001).

**Table 1:** Age, serum progesterone concentrations (P4), estimated time of ovulation and time of laparoscopic intrauterine artificial insemination (IUAI) of bitches with high quality semen (fresh with high spermatozoa concentration) or low quality semen (frozen-thawed with low spermatozoa concentration).

**Bitch ID**	**Age (year)**	**P4 (ng/ml)**	Estimated ovulation time (h)*	Time of laparoscopic IUAI (h)^*^	
1	2	4.6	0	48	FRESH SEMEN (250 x 10^6^ viable spermatozoa)
2	3	1.4	48	96	
3	3	4.1	0	48	
4	3	1.1	48	96	
5	4	2.2	24	72	
6	3	1.5	48	96	
7	2	3.0	24	72	
8	5	3.0	24	72	
9	1.5	6.6	0	48	
10	2	3.4	24	72	
Mean ± SD	2.8±1	3.0±1.6			
11	0.5	1.0	48	96	FROZEN/THAWED SEMEN (80 x 10^6^ viable spermatozoa)
12	2	1.5	48	96	
13	4	2.4	24	72	
14	1	3.3	24	72	
15	4	4.5	0	48	
16	2	2.8	24	72	
17	2.5	3.1	24	72	
18	3	3.9	24	72	
19	5	4.6	0	48	
20	1	1.8	48	96	
Mean ± SD	2.5 ± 1.4	2.8 ± 1.2	-	-	-

ID: identification; SD: standard deviation. *Hours after determination of serum P4 concentration, interval from blood collection to laparoscopic insemination.

### Semen collection and evaluation

Two, clinically healthy, and fertile male dogs (research dogs), aged between 1 and 5 years were used for semen collection. A month previously to the beginning of the study, the animals were conditioned for semen collection by digital manipulation. Semen samples retrieved from both dogs were analyzed and all parameters were considered normal after a complete breeding soundness.

The sperm-rich fraction of the ejaculates, collected by digital manipulation, was analysed for sperm motility, concentration and morphology. Sperm motility was subjectively assessed with a light microscope with a heated stage at 38°C. Sperm cell concentration was determined with a Neubauer haematocytometer chamber and sperm morphology was assessed by Karras staining method modified (Papa et al., 1986).

Only the ejaculates containing a minimum of 80% progressively motile and 80% morphologically normal spermatozoa were used for the experiment.

### Semen freezing and thawing

The sperm was frozen according to the procedure described by Martins (2005). Each semen sample was centrifuged (800 x g) for 10 minutes, and the pellet was suspended in pre-warmed (35-37˚C) extender Tris-glucose-citric acid with 20% egg yolk and 7% (v/v) glycerol (Pena and Linde Forsberg, 2000) to obtain a sperm concentration of 80 x 10^6^/mL. After dilution, the sample was placed in 0.5 mL straws and chilled at 5˚C for 1 hour in a fridge. Then, the straws were exposed for 20 min to nitrogen vapours (6 cm above liquid nitrogen level), before plunging them into liquid nitrogen for storage (Chirinéa et al., 2006).

Four semen straws (total volume of 2 mL), were thawed each time in a water bath at 72˚C for 8 seconds immediately before insemination. Sperm motility was evaluated after thawing and samples containing a minimum of 60% progressively motile spermatozoa were used. Samples were maintained in two syringes (1mL each) in water bath at 37˚C until shortly before the insemination.

### Laparoscopic intrauterine artificial insemination

All bitches underwent a single IUAI 48h after the estimated ovulation time; ten bitches were inseminated with fresh semen and ten with frozen/thawed semen.

The bitches were sedated with 2 mg/kg tramadol (Hipolabor^®^, Belo Horizonte, Brazil) in combination with 1 mg/kg levopromazine (Sanofi Aventis Farmacêutica^®^, Susano, Brazil) injected intramuscularly. After 20 minutes, animals were induced with 5mg/kg propofol (Laboratórios do Brasil^®^, Rio de Janeiro, Brazil), and maintained under inhalant anaesthesia with isofluorane 2.5% (Abbott Laboratories^®^, Abbot Park, IL, USA) diluted in O_2_ (100%).

Bitches were placed in dorsal recumbency in Trendelenburg’s position. A Verres needle (Karl Storz^®^, Tuttlingen, Germany) was inserted through the midline of the abdomen (5 cm caudally to the umbilicus), and a pneumoperitoneum was established with intern abdominal pressure between 8 to 10 mmHg of CO_2._ The Verres needle was then substituted by a camera portal (7 mm trocar) and the laparoscope (Hopkins 26031 H, 7mm, 30cm, 30˚, Karl Storz^®^, Tuttlingen, Germany) was inserted for the initial abdomen inspection. Two portals for insertion of instruments were placed 3 cm caudally to the umbilicus and 2 cm laterally to the first pair of abdominal mammary glands in each side.

The cranial portion of the uterine horns was grasped by forceps and elevated against the ventral abdominal wall. A 22G catheter (BD Angiocath^®^, Juiz de Fora, Brazil) was inserted through the abdominal wall directly into the proximal part of each uterine horn. When reaching the lumen, 1 mL of high quality fresh semen (250 x 10^6^ spermatozoa with 80% motility) or frozen/thawed semen (80 x 10^6^ spermatozoa with 60% motility) loaded into a syringe, was injected in each horn. The location of the catheter into the lumen was confirmed by the increased diameter of the uterine horn while the sample was injected. Inspection of abdominal cavity was performed before removing instruments and the pneumoperitoneum was deflated. Incisions were sutured conventionally in two layers (abdominal fascia and skin). Analgesia was given during post operatory period.

### Recovery rates, pregnancy rates and in vivo early embryonic development

Embryos or unfertilized oocytes were collected 7 days after insemination (or 9 days of presumed ovulation) (Pretzer, 2008), once the authors aimed to verify the absence or presence of embryos at early stages of development. At this time a conventional ovariohysterectomy was performed, being the uterine body excised immediately cranial to the cervix. Ovaries were macroscopically examined and the number of corpora lutea (CL) was recorded. Oviducts and uterine horns were excised above a collecting recipient and flushed separately with 10 mL of PBS (Dulbecco’s Phosphate Buffered Saline) supplemented with 10% FBS (Fetal Bovine Serum) in order to recover embryos and unfertilized oocytes.

Prior to the uterine tube flushing, the uterine horns were sectioned immediately distal to uterotubal junction and uterine bifurcation. Oviducts flushing was performed with the insertion of a 19 G plastic catheter into the lumen. For the uterine horns and uterine body a 22G catheter was used (Tsutsui et al., 2001) and these structures were flushed in different sessions, separately.

Pregnancy rate was estimated as the number of bitches from which the embryos were recovered out of the total number of bitches inseminated. Recovery rates were calculated considering the number of collected embryos and the number of CL macroscopically observed on the ovarian surface of individual bitches (Walter et al., 2011).

Collected embryos were evaluated at the stereomicroscope and assessed for symmetry, blastomere shape and darkness uniformity. Embryos of good to excellent quality (grade 1 or 2) were perfectly symmetrical (or only slightly asymmetrical), spherical, and uniformly dark (Bo and Mapletoft, 2013). Embryos were classified as degenerated when pale in colour or with lysed blastomeres. Degeneration rate was derived by the number of degenerated embryos on the total number of recovered embryos.

To assess the embryonic developmental stage and the nuclear stage of unfertilized oocytes, embryos or oocytes were stained with bis-benzimide (Hoechst 33342) and propidium iodide and examined under a fluorescence microscope (Olympus IX 70, Olympus America Inc^®^, Center Valley, PA, USA), using the appropriate filter.

### Statistical analysis

Results reported as mean ± standard deviation were analyzed using SAS system (Statistical Analysis System). Correlations between selected variables (number of CL, progesterone concentrations, number of embryos and oocytes) were evaluated using Spearman correlation test. Differences between the mean number of embryos or oocytes recovered in oviducts compared to uterine horns, and pregnancy rates in different groups of bitches were evaluated by ANOVA. Results were considered significant when P <0.05.

## Results

### Estimation of ovulation time and time of laparoscopic IUAI

Serum concentrations of progesterone when 90% of vaginal cells were cornified ranged from 1.0 to 6.6 ng/mL with an average of 3.0±1.5 ng/mL. As previously mentioned, the day to perform the IUAI with fresh or frozen/thawed semen (Table 1) was determined on the estimation of ovulation time derived by one measurement of progesterone concentration.

### Recovery rates, pregnancy rates and in vivo embryo development

The individual embryo recovery rates (n. embryos/n. CL) ranged from 0% to 75% with an average value of 20 ± 22.7% when high quality fresh semen was used (Table 2) and from 0% to 40% with an average value of 8.7 ± 13.5% (Table 3) when bitches were inseminated with low quality semen (frozen/thawed). After flushing of oviducts and uterine horns, 20 embryos were recovered from 8 out of 10 bitches (80% pregnancy rate) with a value ranging from 1 to 6 embryos per bitch inseminated with fresh semen. Inseminations with thawed/frozen semen resulted in 10 embryos (from 1 to 4 in each bitch) recovered in 5 out of 10 bitches (50% pregnancy rate). Non-fertilised oocytes were retrieved on animals that provided embryos or not (Table 2, Table 3).

**Table 2:** Recovery rates, stage, site of recovery and degeneration rates of embryos and oocytes recovered 7 days after laparoscopic intrauterine artificial insemination of bitches with high quality semen (fresh with high spermatozoa concentration).

**Bitch ID**	**Recovery rates^§^ %**	**Total CL n.**	**Total Embryos n.**	**Embryo stage**	**Embryo degeneration n. (%)**	**Embryos in uterine tubes n.**	**Embryos in uterine horns n.**	**Total Oocytes n.**	**Oocyte meiotic** **stage**	**Oocyte degeneration** **n. (%)**	**Oocytes in uterine** **tubes n.**	**Oocytes in uterine horns n.**
1	14.3	14 (8R 6L)	2	4 cells	0 (0)	2 (1R 1L)	-	6^§§^	1 MI; 3 MII§§	2 (33.3)	4 (4R 0L)	2 (0R 2L)
2	0	12 (7R 5L)	0	-	-	-	-	2	2 MII	0 (0)	2 (1R 1L)	-
3	0	12 (5R 7L)	0	-	-	-	-	3	2 MI	1 (33.3)	3 (0R 3L)	
4	7.1	14 (9R 5L)	1	4 cells	0 (0)	1 (1R 0L)	-	-	-	-	-	-
5	7.1	14 (8R 6L)	1	4/6 cells	0 (0)	1 (0R 1L)	-	-	-	-	-	-
6	75.0	8 (5R 3L)	6	8 cells	2 (33.3)	6 (4R 2L)	-	-	-	-	-	-
7	20.0	5 (2R 3L)	1	3 cells	0 (0)	-	1 (0R 1L)	-	-	-		-
8	30.0	10 (5R 5L)	3	4 cells	0 (0)	3 (2R 1L)	-	-	-	-	-	-
9	35.7	14 (8R 6L)	5	4/6 cells	1 (20)	5 (2R 3L)	-	-	-	-	-	-
10	11.1	9 (5R 4L)	1	4 cells	0 (0)	-	1 (0R 1L)	-	-	-	-	-
Total	-	112 (62R 50L)	20	-	3	18 (10R 8L)	2 (0R 2L)	11	-	3 (27.3)	9 (5R 4L)	2 (0R 2L)
Mean ± SD	20.0 ± 22.7	11.2 ± 3.1	2.0 ± 2.1	-	0.3 ± 0.7	1.8 ± 2.2*	0.2 ± 0.4**	1.1 ± 2.0	-	0.3 ± 0.7	0.9 ± 1.5	0.2 ± 0.6

ID: identification; MI: metaphase I; MII: metaphase II; SD: standard deviation. §Recovery rates: n. embryos/n. corpora lutea (CL); §§All oocytes in the oviducts a part of except for 1 MI and 1 MII in uterine horns. */**Superscripts indicate significant differences P<0.05.

**Table 3:** Recovery rates, stage, site of recovery and degeneration rates of embryos and oocytes recovered 7 days after laparoscopic intrauterine artificial insemination of bitches with low quality semen (frozen-thawed with low spermatozoa concentration).

**Bitch ID**	**Recovery rates** **§** **%**	**Total CL n.**	**Total Embryos n.**	**Embryo stage**	**Embryo degeneration n. (%)**	**Embryos in uterine tubes n.**	**Embryos in uterine horns n.**	**Total Oocytes n.**	**Oocyte meiotic** **stage**	**Oocyte degeneration** **n. (%)**	**Oocytes in uterine** **tubes n.**	**Oocytes in uterine horns n.**	
11	0	10 (7R 3L)	0	-	-	-	-	6§§	1MI, 2 MII§§	3 (50)	4 (4R 0L)	2 (0R 2L)
12	10.0	10 (5R 5L)	1	4 cells	0 (0)	1 (1R 0L)	-	0				-
13	0	7 (4R 3L)	0	-	-	-	-	3	2 MII	1 (33.3)	2 (2R 0L)	1 (0R 1L)
14	18.2	11 (7R 4L)	2	4 cells	0 (0)	2 (1R 1L)	-	0	-	-	-	-
15	10.0	10 (6R 4L)	1	2 cells	0 (0)	1 (0R 1L)	-	0	-	-	-	-
16	0	9 (6R 3L)	0	-	-	-	-	3	1 MI, 1MII	1 (33.3)	3 (0R 3L)	-
17	40.0	5 (2R 3L)	2	4 cells	0 (0)	2 (2R 0L)	-	0	-	-	-	-
18	0	8 (5R 3L)	0	-	-	-	-	4	3 MII	1 (25)	3 (2R 1L)	1 (1R 0L)
19	28.6	14 (9R 5L)	4	4 cells	0 (0)	3 (2R 1L)	1 (1R 0L)	0	-	-	-	-
20	0	9 (5R 4L)	0	-	-	-	-	1	1 MII	-	1 (1R 0L)	-
Total	-	93 (56R 37L)	10	-	0	9 (6R 3L)	1 (1R 0L)	17	-	6 (35.3)	13 (9R 4L)	4 (1R 3L)
Mean ± SD	8.7 ± 13.5	9.3 ± 2.4	1.0 ± 1.3	-	-	0.9 ± 1.1*	0.1± 0.3**	1.7 ± 2.2	-	0.6 ± 1.0	1.3 ± 1.6	0.4 ± 0.7

ID: identification; MI: metaphase I; MII: metaphase II; SD: standard deviation. §Recovery rates: n. embryos/n. corpora lutea (CL); §§All oocytes in the oviducts excepting1 MI and 1 MII in uterine horns . */**Superscripts indicate significant differences P<0.05.

More embryos were recovered from the flushing of the oviducts compared to the uterine horns, both for the fresh and frozen/thawed semen groups (1.8 ± 2.2 vs 0.2 ± 0.4; P<0.05 and 0.9± 1.1 vs. 0.1± 0.3; P<0.05, respectively. Table 2, Table 3). All the non-degenerated embryos ranged from 2 to 8-cells stage were classified as grade I according to their morphology. Degeneration rates were low (ranging from 0 to 0.3 ± 0.7 for frozen/thawed and fresh semen, respectively), and the embryos found in each bitch were at the same stage, even though different stages of embryo development were observed among the bitches.

The majority of unfertilized oocytes collected from the oviducts or horns was morphologically normal at a meiotic stage ranging from metaphase I and II, being surrounded by more than three layers of compact cumulus cells. Some of them had the external layer, less attached and expanded, but none was devoid of cumulus cells. No embryos/oocytes were recovered from uterine bodies that were flushed separately from uterine horns.

Corpora lutea were almost equally distributed in the right and left ovary (Table 2, Table3), but no significant correlations were found between number of embryos/oocytes recovered from one side of oviduct/horn and number of CL observed in ipsilateral the ovary, and between total number of embryo/oocytes and number of CL or progesterone concentrations. The number of CL also did not correlate significantly with progesterone concentrations.

Recovery and pregnancy rates of bitches grouped according to progesterone concentration and related time of IUAI did not show statistical differences neither for fresh, nor for frozen/thawed semen (Table 4, Table 5).

**Table 4:** Recovery and pregnancy rates in bitches categorized according to progesterone (P4) concentration and related time of laparoscopic intrauterine artificial insemination (IUAI) with high quality semen (fresh with high spermatozoa concentration).

	**Bitch ID**	**Embryos n.**	**Corpora lutea n.**	**Recovery rates** **§** **%**	**Pregnancy rates** **§§** **n. (%)**
P4 >1 - 1.9 ng/ml IUAI 96h later	2	0	12	0	2/3 (66.7)
	4	1	14	7.1	
	6	6	8	75.0	
Total		7	34		
Mean ± SD		2.3 ± 3.2	11.3 ± 3.1	27.4 ± 41.4	
P4 2 - 3.9 ng/ml IUAI 72h later	5	1	14	7.1	4/4 (100.0)
	7	1	5	20.0	
	8	3	10	30.0	
	10	1	9	11.1	
Total		6	38		
Mean ± SD		1.5 ± 1.0	9.5 ± 3.7	17.1 ± 10.2	
P4 4 -10 ng/ml IUAI 48 h later	1	2	14	14.3	2/3 (66.7)
	3	0	12	0	
	9	5	14	35.7	
Total		7	40		
Mean ± SD		2.3 ± 2.5	13.3 ± 1.2	16.7 ± 18.0	

ID: identification; SD: standard deviation. No significant differences were observed within columns. §Recovery rates: n. embryos/n. corpora lutea; §§Pregnancy rates: n. bitches from which the embryos were recovered/total number of bitches inseminated.

**Table 5:** Recovery and pregnancy rates in bitches categorized according to progesterone (P4) concentration and related time of laparoscopic intrauterine artificial insemination (IUAI) with low quality semen (frozen-thawed with low spermatozoa concentration).

	**Bitch ID**	**Embryos n.**	**Corpora lutea n.**	**Recovery rates** **§** **%**	**Pregnancy rates** **§§** **n. (%)**
P4 >1 - 1.9 ng/ml IUAI 96h later	11	0	10	0	1/3 (33.3)
	12	1	10	10.0	
	20	0	9	0	
Total		1	29		
Mean ± SD		0.3 ± 0.6	9.7 ± 0.6	3.3± 5.8	
P4 2 - 3.9 ng/ml IUAI 72h later	13	0	7	0	2/5 (40.0)
	14	2	11	18.2	
	16	0	9	0	
	17	2	5	40.0	
	18	0	8	0	
Total		4	40		
Mean ± SD		0.8 ± 1.1	8.0 ± 2.2	11.6 ± 17.7	
P4 4 -10 ng/ml IUAI 48 h later	15	1	10	10.0	2/2 (100.0)
	19	4	14	28.6	
Total		5	24		
Mean ± SD		2.5 ± 2.1	12.0 ± 2.8	19.3 ± 13.2	

ID: identification; SD: standard deviation. No significant differences were observed within columns. §Recovery rates: n. embryos/n. corpora lutea; §§Pregnancy rates: n. bitches from which the embryos were recovered/total number of bitches inseminated.

## Discussion

Present data show that a single laparoscopic insemination performed 48 h after the ovulation, estimated by a single progesterone determination performed when the vaginal cytology showed 90% cornified cells, resulted respectively in 80% and 50% pregnancy rate in bitches when high quality semen (fresh and with high concentration) or low quality semen (frozen/thawed with low concentration) were used.

These results indicate that, although the evaluation of ovulation time was not as precise as with serial progesterone determinations, in case of logistic or economic limitations, which prevent a more accurate estrous monitoring, pregnancies can be obtained especially with high quality semen, although with a variable number of embryos (ranging from 1 to 6). This result can be usefull to breeders that are interest to achieve pregnancies even with fewer foetus and the risk of singleton pregnancy driving to dystocia.

The pregnancy rate of 80% is comparable to the obtained in a study using serial determinations of progesterone and three IUAI with highly concentrated fresh semen were performed (90%) [8] or in other reports where only a single IUAI was used (84%) (Burgess et al., 2012); 72% (Gaytan et al., 2020). Concerning frozen semen higher percentages of pregnancy (78-100%) were previously obtained (Silva and Verstegen, 1995; Tsutsui et al., 2000; Mason and Rous 2014) compared to the 50% of the present work, but in all cases the right time of insemination (laparoscopical, endoscopical or surgical) was evaluated with serial progesterone determinations and a high semen concentration (ranging from >150 x 10^6^ spermatozoa to 1 x 10^8^ spermatozoa) was used.

It is interesting to note that grouping the bitches according to progesterone concentration and related time of IUAI (P4 1-1.9 ng/mL, IUAI at 96h; P4 2-3.4 ng/mL, IUAI 72h; P4 4-10 ng/mL, IUAI 48h), recovery and pregnancy rates were similar between groups when either fresh or frozen semen was used. This means that, although low concentrations of P4 could have been less indicative and more prone to errors in planning insemination time, laparoscopic insemination with high or low quality semen resulted in pregnancy rates comparable to those described in studies where a more accurate estrous monitoring was performed (Mason 2017; Mason and Rous, 2014; Macedo et al., 2012; Tsutsui et al., 2000; Silva and Verstegen, 1995).

In the present study bitches were inseminated only once since laparoscopy, although it is a minimally invasive technique, it should not be repeated due to the trauma and surgical stress. Semen deposition in both uterine horns may not be as efficient as increasing the number of inseminations to effectively enhance the chances of oocyte fertilization, however multiple laparoscopic insemination is not a good alternative due to the anaesthetic and surgical risks. The use of higher insemination doses in a single IAIU could contribute to enhance chances of oocyte fertilization, once a greater number of viable sperm would be present in the female genital tract, and spermatozoa from a good quality fresh or post-thawed semen are viable 2 to 4 days after ovulation time (Karre et al., 2012). However, if there is a correlation between high insemination doses and increased fertility, it remains to be investigated.

Spermatozoa distribution occurred evenly along both horns, regardless of the site of semen deposition, as demonstrated with laparoscopical insemination in the uterine body or in the cranial tip of one uterine horn (Fukushima et al., 2010)

Present data showed that recovery rate (n. embryos/n. CL) was very variable (range 0 to 75% for fresh semen and 0 to 40% for frozen semen). These results are low when compared with the average of 63%-71% obtained in other studies (Commin et al., 2012; Miranda et al., 2018). However, it is difficult to compare data of the literature that were obtained after an accurate evaluation of day 0 (day of LH surge) with serial progesterone determinations and with 2-3 inseminations with fresh semen (Commin et al., 2012; Miranda et al., 2018), with those of the present work in which the single progesterone determination was not as accurate in predicting the optimal time for insemination. Additional progesterone determinations can be considered, however additional costs must be taken into account.

The total number of CL did not correspond to the sum of retrieved embryos and oocytes and no significant correlations were found, while others reported that number of CL was highly correlated with the number of recovered embryos (Miranda et al., 2018). The reasons of this high variability of recovery rates could be either the loss of embryos/oocytes during dissection of the organ and particularly during the flushing of the oviducts, or the occurrence of early resorption of embryos, as described by other authors (Tsutsui et al., 2009; Verstegen et al., 2008).

Significant correlation has not even been found between number of oocytes/embryos recovered from each oviduct and uterine horn, and the side of the ovarian CL. This finding could have little relevance either because of the low number of oocytes/embryos recovered or by the CL formed, existing migration of embryos along the two horns reported for the preimplantation period (9-12 days after LH surge) (Pretzer, 2008), transuterine migration of embryos occurs due to the number of embryos that enter the uterus and there are no reports about the transuterine migration of unfertilized oocytes (Tsutsui et al., 2002).

One to six grade I embryos were recovered from each pregnant bitch; most of the embryos at 2 to 8-cells stage were retrieved from the oviducts and few from the uterine horns at 7 days after the laparoscopic insemination. Recent data reported (Miranda et al., 2018) showed that embryos at different stages (2 to 16 cells, and even morula) were recovered until day 10 following the pre-ovulatory LH surge either from the oviducts or the uterus, confirming, as previously shown (Bysted et al., 2001), that non-synchronous ovulations occur, but also suggesting that embryo entrance into the uterus does not depend on the developmental stage.

These data are hardly comparable with those of the present work since the intravaginal insemination with fresh semen in a wide time interval (between 3 to 6 days after LH surge) and the recovery between 8 and 11 days after the LH surge (accurately determined) may have contributed to the variety of embryo developmental stages at recovery. The occurrence of a non-synchronous ovulation could not be proved with the present data, because of the low recovery rates and because recovered embryos were at the same stage in the same bitch. However, poor synchronization between oocyte maturation and insemination resulting in late fertilization could have also resulted in fewer early stage embryos predominately located in the oviducts (Commin et al., 2012; Miranda et al., 2018). Another reason of the lower variability of embryo stages at recovery might be the reduced fertilization time due to use of single insemination close to the site of fertilization, compared to the studies in which the time of fertilization was extended because several inseminations were performed. Additionally, the lower variability of embryo stages can be due to the time of sperm survival from frozen/thawed semen that could be shorter than fresh sperm due to the damage caused by the freezing process. That fertilization occurs 48-83 h after ovulation (and embryos at 2-cell stage were observed at 4-7 days after ovulation, while embryos at 8-cell-stage developed 4.5-12 days after ovulation (Chastant-Maillard et al., 2010; Reynaud et al., 2005).

The majority of unfertilized oocytes was collected from oviducts rather than from uterine horns and their morphology was good according to the characteristics previously reported (Reynaud et al., 2005).

Few studies exist on the meiotic resumption stage of oocytes matured *in vivo*. In the present study, oocytes harvested 9 days after the estimated ovulation were at the MI and MII stage of the meiosis. These results differ from previous data (Reynaud et al., 2005) which hypothesized an asynchrony of follicle ovulation and a diversity between maturation stages of oocytes collected from oviducts. However, in their study, ovulation was accurately determined with serial ultrasonographic examinations of the ovaries and bitches were ovariohysterectomized at different time intervals from ovulation (from 15 to 136 h) in order to record the first appearance of MI/MII stage oocytes. In the present study, the recovery of early stages embryos mainly in the oviducts, the low degeneration rates, the meiotic stages of unfertilized oocytes that were also recovered from the oviducts rather from the uterine horns, suggest that the actual ovulation occurred later than the estimated ovulation day. A recovery from uterus at 8-11 days after the LH surge resulted in the retrieval of unfertilized oocytes (Miranda et al., 2018) and supported the finding that oocytes, other than embryos, migrate into the uterus 7-10 days after ovulation [18]. In this study, the absence of embryo/oocytes on the uterine body can be associated to probable loss during recovery. In accordance with previous reports, uterine glands, uterine crypts and the utero-oviduct junction are the main sites for sperm storage in the dog (Karre et al., 2012). Thus, probable embryo storage on these sites during the flushing procedure should be considered. No significant correlations were found between progesterone concentrations and the number of CL, or number of recovered embryos/oocytes. This finding could confirm that plasma concentrations of progesterone during pregnancy are independent of the number of CL Miranda et al., 2018), but the low recovery rate could have affected the correlation with the number of embryos/oocytes.

## Conclusion

A laparoscopic intrauterine insemination, based on a single progesterone determination, can result in pregnancies with both high quality (fresh high sperm concentration) or low quality (frozen/thawed low sperm concentration) semen, although with a low number of embryos. Embryos recovered at early developmental stages mainly from the oviducts suggest that ovulation might have occurred later than expected even in those bitches in which progesterone concentration was higher (4-10 ng/mL) and therefore potentially more indicative for the estimation of ovulation time.

This observation should be taken into account in case economic or logistic restrictions affect the possibility of owners to plan an accurate monitoring of the optimal breeding time.
